# Advanced sporadic renal epithelioid angiomyolipoma: case report of an extraordinary response to sirolimus linked to *TSC2* mutation

**DOI:** 10.1186/s12885-018-4467-6

**Published:** 2018-05-15

**Authors:** Marta Espinosa, Juan Maria Roldán-Romero, Ignacio Duran, Enrique de Álava, María Apellaniz-Ruiz, Alberto Cascón, Carmen Garrigos, Mercedes Robledo, Cristina Rodriguez-Antona

**Affiliations:** 1 Medical Oncology Department, Hospital Virgen del Rocío, Servicio de Oncología Medica, Avenida Manuel Siurot s/n, 41013 Sevilla, Spain; 2 Hereditary Endocrine Cancer Group, Human Cancer Genetics Programme, Spanish National Cancer Research Centre (CNIO), Melchor Fernández Almagro 3, 28029 Madrid, Spain; 3 Instituto de Biomedicina de Sevilla, IBiS/Hospital Universitario Virgen del Rocío/CSIC/Universidad de Sevilla, Sevilla, Spain; 4 Pathology Department, Instituto de Biomedicina de Sevilla, IBiS/Hospital Universitario Virgen del Rocío/CSIC/Universidad de Sevilla-CIBERONC, Sevilla, Spain; 5 Centro de Investigación Biomédica en Red de Enfermedades Raras (CIBERER), Valencia, Spain

**Keywords:** Renal epithelioid angiomyolipoma, Sirolimus, TSC2 mutation, mTOR pathway activation

## Abstract

**Background:**

Renal epithelioid angiomyolipomas (EAML) are rare tumors with aggressive behavior. EAML can be sporadic or develop within the tuberous sclerosis complex syndrome, where mutations of *TSC1* or *TSC2* genes (critical negative regulators of mTOR Complex 1) result in an increased activation of mTOR pathway. Optimal EAML treatment, including mTOR inhibitors, remains undetermined.

**Case presentation:**

Here we present the case of a young adult with a renal EAML that after radical nephrectomy developed metastases, first in liver and then in lumbar vertebrae. After complete surgical resection of these lesions, liver recurrence was detected, this time with incomplete surgical resection. After finding a new liver lesion, systemic treatment with sirolimus started. The patient exhibited a complete and durable response to this drug, being disease free at the time of publication, after 36 months of treatment. Targeted next generation sequencing (NGS) of *MTOR*, *TSC1* and *TSC2* genes in the primary tumor, metastasis and blood of the patient, revealed one inactivating *TSC2* mutation (c.2739dup; p.K914*) in the tumor cells. Immunohistochemistry revealed decreased TSC2 protein content and increased phospho-S6 in the tumor cells, demonstrating mTOR pathway activation.

**Conclusion:**

NGS on an EAML patient with an extraordinary response to sirolimus uncovered *TSC2* inactivation as the mechanism for the response. This study supports NGS as a useful tool to identify patients sensitive to mTOR inhibitors and supports the treatment of malignant EAML with these drugs.

## Background

Angiomyolipomas (AML) are rare kidney tumors that occur in 0.2–0.3% of the population [1, 2]. These neoplasms are mesenchymal in origin and comprise blood vessels, mature adipose tissue and fusiform cells similar to smooth muscle. Depending on the major component, AML is histologically classified into fat-predominant, smooth muscle-predominant, epithelioid, oncocytic and sclerosant subtype [3]. Most AMLs are considered benign and have a primarily local growth. However, one particular subtype characterized by the presence of an epithelioid cellular morphology, named epithelioid AML (EAML), and included in the family of perivascular epithelioid cell tumors (PEComas) can have malignant behaviour [4, 5]. Histologically, EAML cells contain granular cytoplasm in more than 5% of tumor volume, with round nucleus and occasional multinucleated giant cells dispersed. They can co-express melanocytic and muscle markers and are negative for epithelial markers. The low percentage of fat is characteristic of this variety of AML and makes diagnosis difficult with CT or MRI.

The behaviour of EAML is variable, ranging from indolent, with only local growth, to aggressive, with potential for invasive growth and dissemination. This different behaviour seems to be determined by the presence or absence of cellular atypia and other clinical and pathological factors. The definition of atypical epithelioid cells in series of EAML includes atypical polygonal cells with abundant cytoplasm, vesicular nuclei, prominent nucleoli and nuclear size that exceeds twice the size of the adjacent nuclei. In a comparative analysis of the literature published by Brimo et al., 21 EAML cases with benign clinical course were compared with 9 EAML with malignant behaviour [6]. The aggressive cases tended to associate with older patients, larger tumor size, higher percentage of epithelioid component, severe atypia, higher percentage of atypical cells, higher mitotic count, atypical mitotic figures, necrosis, lymphovascular invasion, and renal vein invasion. A predictive model was developed that included the following factors: i) ≥ 2 mitotic figures per 10 high-power fields; ii) ≥ 70% atypical epithelioid cells; iii) atypical mitotic figures; iv) necrosis. The presence of 3 or more of these factors was highly predictive of malignant behaviour [6]. Moreover, the evaluation of another series of 41 EAML patients identified a number of clinico-pathologic parameters that also predicted for worse outcome including: i) tuberous sclerosis complex (TSC) or concurrent AML; ii) necrosis; iii) tumor size> 7 cm, iv) extra renal extension and/or vein involvement, v) carcinoma-like growth pattern. Based on these findings the authors suggested that EAML patients should be classified in groups with low, intermediate and high risk of disease progression, according to the presence of 0–1, 2–3 or 4–5 of these parameters, respectively [7].

Approximately 80% of AML are sporadic while 20% develop within the TSC [8]. AML tumors in the context of TSC present bi-allelic inactivating mutations in *TSC1* or *TSC2*. These genes encode the proteins hamartin and tuberin responsible for the inhibition of the mammalian target of rapamycin (mTOR), a conserved protein kinase that regulates cell growth and metabolism in response to growth factors and nutrients [9]. Rapamycin and its analogs, sirolimus, everolimus and temsirolimus, inhibit mTOR pathway and are anti-tumor drugs used for metastatic renal cell carcinoma, pancreatic neuroendocrine tumors and advanced breast cancer. In addition, everolimus has shown significant efficacy to treat TSC neoplasms, including AML [10]. Significant clinical response to mTOR inhibitors has been described in patients with unresectable or recurrent PEComas, although specific data regarding EAML is scarce [11, 12]. Whenever possible, treatment of EAML should be surgery. Chemotherapy has limited benefit, while response to mTOR inhibitors, awaiting clinical trials, remains undetermined with only isolated cases reported in the literature with contradictory outcomes [13–16].

In this study we performed a genomic and immunohistochemical characterization of an EAML patient that after developing hepatic and bone metastasis had a complete response to sirolimus, and 36 months after the start of treatment remains disease-free. The molecular mechanism responsible for this extraordinary response to sirolimus was identified by next generation sequencing (NGS) and immunohistochemistry (IHC).

## Case presentation

**Fig. 1:**
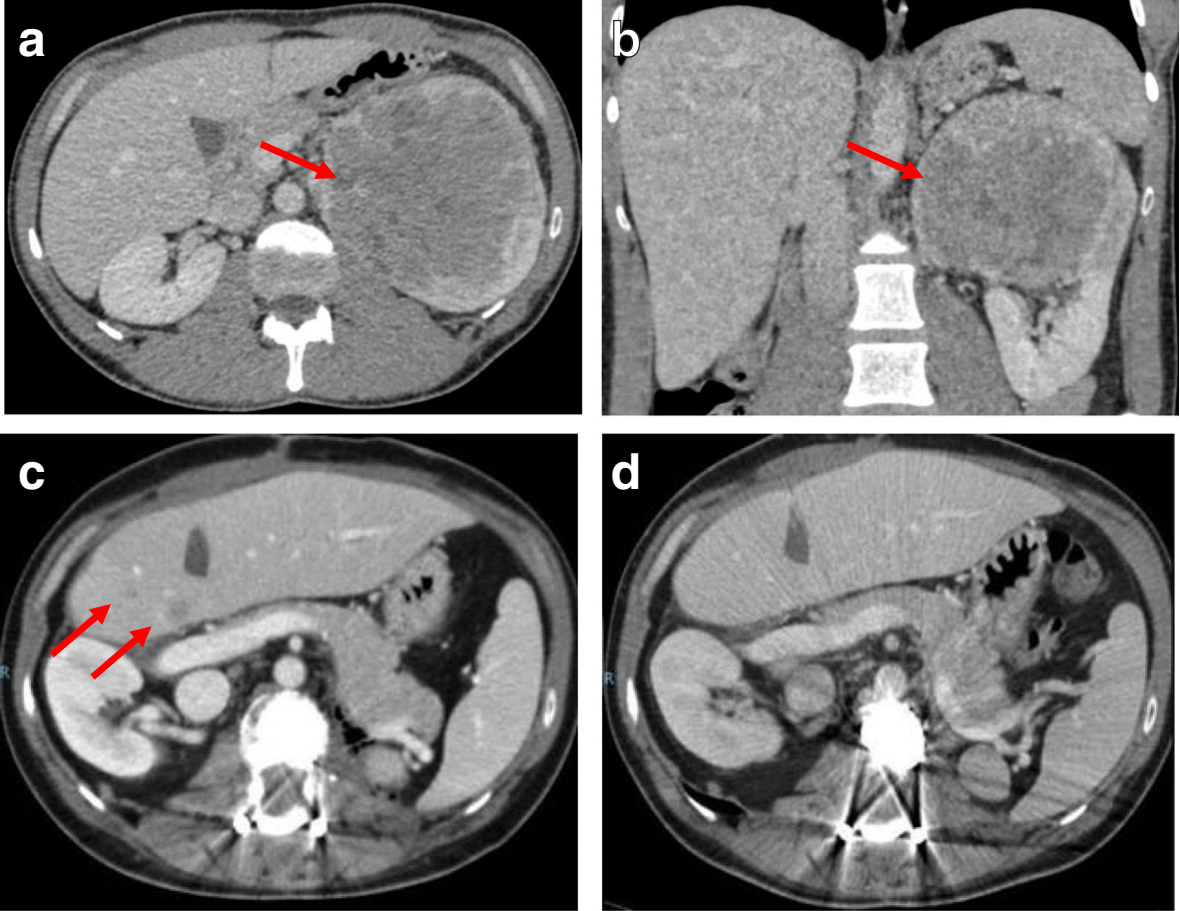
Computed tomographic (CT) scans. Left renal mass of 10 × 12 cm at diagnosis (**a** and **b**). Liver recurrence (**c**) and response after 5 months of sirolimus treatment (**d**)

**Fig. 2:**
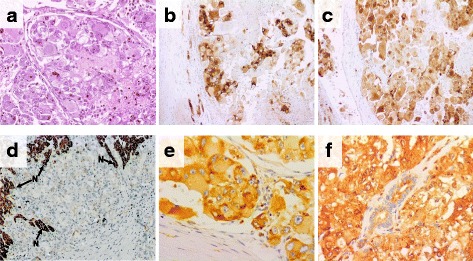
Immunohistochemical study. Hematoxylin and eosin staining of the EAML primary tumor (**a**). Representative images for HMB-45 (**b**; × 10) and Melan A (**c**; × 10). TSC2 staining (Cell Signalling 4308) of the tumor metastasis, where tumor cells are negative for TSC2 while normal hepatocytes (indicated with an arrow and “N”) show high intensity. (**d**; 10×). Phospho-ribosomal protein S6 (S235/S236; Cell Signaling 2211) expression in the primary tumor (**e**; × 40) and liver metastasis (**f**; × 10)

We report the case of a Caucasian male patient aged 34 without an irrelevant past medical history that presented with unexplained weight loss. Imaging studies revealed the presence of a left renal mass of 10 × 12 cm (Fig. 1a-b). The patient underwent a left radical nephrectomy and the pathology was consistent with an EAML (Fig. 2a) with poor prognosis features (size > 7 cm, vascular and renal sinus invasion, necrosis, and severe atypia). Immunohistochemical profile revealed diffuse and intense expression of HMB-45 and Melan A (Fig. 2b-c), along with expression of smooth muscle actin and CD68 (KP-1, Ventana), and negativity for CD-31, CEA, CK-pan, desmin, EMA, Ki-67, myogenin and S-100. After nephrectomy the patient did not receive adjuvant therapy and started follow up in urology clinics. Seven months after primary surgery the patient developed 3 liver metastases (two in segment VIII of 6 cm and 1.5 cm, respectively, and one in segment IV of 2 cm). All these lesions were completely resected through a right partial hepatectomy with extension to segment IV through a split in situ technique. Again, adjuvant therapy was not administered. Five months later a new single metastasis developed in the first lumbar vertebrae, and was managed through a total L1 corpectomy. Six months after the spine surgery liver recurrence was observed, surgical resection was incomplete leaving positive margins and within 12 weeks a new liver lesion of 1.7 cm was detected. A new surgical attempt was considered not feasible and after reviewing the scarce existing literature, it was decided to start systemic treatment with sirolimus 6 mg/day [13]. After starting sirolimus treatment the patient presented a very unusual and favorable response (complete response after 13 months of treatment; Fig. 1c-d). Tolerance was excellent with grade 1 intermittent diarrhea and acne along with grade 1 hypophosphatemia. At the time of publication, after 36 months of treatment and about five years of the initial diagnosis, the patient remains free of disease and with an excellent performance status. Genetic testing was performed and ruled out TSC (no *TSC1* or *TSC2* germline mutations detected).

**Fig. 3:**
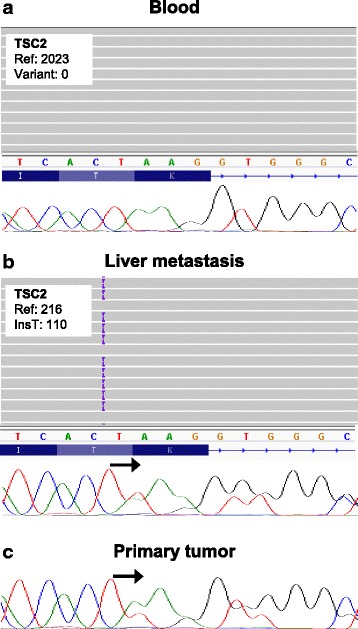
Tumor *TSC2* point mutation. Targeted NGS revealed one frameshift mutation in *TSC2* gene (c.2739dup; p.K914*) absent in the patient’s blood (**a**) and present in the liver metastasis (**b**). The primary tumor DNA failed NGS technique, but Sanger sequencing detected the *TSC2* mutation (**c**). Representative genome images from the Integrative Genomics Viewer (Broad Institute) are shown together with Sanger chromatograms

Targeted NGS of *MTOR*, *TSC1* and *TSC2* genes was performed on DNA extracted from formalin-fixed paraffin-embedded primary tumor and hepatic metastasis, and the patient’s peripheral blood (TruSeq Custom Amplicon Low Input; Illumina). Primary tumor failed NGS due to poor DNA quality, however, the liver metastasis and the blood were successfully sequenced by NGS, with a mean coverage of 184× and 1643×, respectively, and single nucleotide variants and indels were identified. One *TSC2* variant resulting in a premature stop codon (c.2739dup; p.K914*) was found in heterozygosity in the metastasis while it was absent in blood (Fig. 3a-b). Sanger sequencing validated this finding, and detected the *TSC2* mutation also in the primary tumor (Fig. 3c). IHC revealed absence of TSC2 expression in the liver metastasis (Fig. 2d), in agreement with inactivation of *TSC2*. Phospho-ribosomal protein S6 staining was positive in the primary tumor and liver metastasis (Fig. 2e-f), indicating activation of the mTOR pathway in the patient’s tumors.

## Discussion and conclusions

mTOR signaling pathway is up-regulated in many cancers and hamartoma syndromes through mutations in genes that participate in this pathway. Genetic events include activating mutations in *MTOR* and *PIK3CA* and inactivating mutations in *TSC1*, *TSC2* and *PTEN*. Extraordinary responses to mTOR inhibitors are rare and have been described in patients with metastatic bladder cancer [17, 18], and in an anaplastic thyroid cancer patient [19]. In these cases, mutations in *TSC1*, *TSC2* or *MTOR* were identified as the mechanism leading to the drug sensitivity. However, a recent study in renal cell carcinoma showed that some patients with mutations activating mTOR pathway did not respond to mTOR inhibitors, while some without mutations did [20], suggesting that tumor specific mechanisms may be modulating response. Thus, further investigation and cases with extraordinary responses are required to understand the mechanisms responsible for the sensitivity to mTOR inhibitors.

Genetic studies have shown that AML occur due to bi-allelic inactivation of either *TSC2* or *TSC1* [21]. In the case of TSC AML it is caused by a germline mutation in either of these genes plus a tumor second hit, while sporadic AML is almost exclusively caused by mutations in *TSC2* [22, 23]. In both cases hyperactivation of mTORC1 occurs, leading to tumor development. In EAML, similarly to AML, *TSC2* gene deletions seem to be frequent [24], however, studies are scarce. The EAML patient presented here is a sporadic case with a novel *TSC2* mutation (c.2739dup, p.K914*), not described previously in COSMIC or in the germline TCS2 Leiden Open Variation Database (LOVD). However, the LOVD includes a sporadic TSC patient with a TSC2 protein truncated at Threonine 913 (c.2737_2738delAC), allowing to classify this novel variant as pathogenic. Inactivation of *TSC2* and over-activation of mTORC1 in the tumor cells were confirmed by IHC, and were in agreement with the extraordinary response to sirolimus.

Previous studies have reported mTOR pathway activation for *TSC1/TSC2* mutations in sporadic AML and PEComas [25–27], suggesting that mTOR inhibition could potentially provide a therapeutic benefit. A double-blind, placebo-controlled, phase 3 trial tested the efficacy of the mTOR inhibitor everolimus in patients with AML associated with TSC or sporadic lymphangioleiomyomatosis. The study showed that everolimus was superior to placebo and reduced significantly AML volume with an acceptable safety profile, in 2012 this drug was approved for the treatment of adults with renal AML associated with TSC who do not require immediate surgery [28].

However, therapeutic experience with aggressive EAML is scarce. There are only about a dozen published cases in the literature in which EAML patients received mTOR inhibitors with variable outcomes. These reports consist on clinical descriptions that include favorable responses, in most of the cases with no genetic study associated [13, 14, 29–31]. Additionally, other publications have reported unsatisfactory responses to mTOR inhibition [15, 16], suggesting differences in the driver pathways of these tumors.

In summary, only a few cases of EAML with benefit from mTOR inhibitors have been reported, and the mechanisms underlying these responses are unexplored. This study reveals *TSC2* deficiency in a sporadic EAML patient as the mutation causative of an exceptional response to sirolimus. These results support NGS as a useful tool to predict sensitivity to mTOR inhibitors in patients with EAML, this rare and potentially aggressive urological malignancy is misrepresented in clinical trials.

## Abbreviations

AML: Angiomyolipomas
EAML: Epithelioid angiomyolipomas
IHC: Immunohistochemistry
mTOR: Mammalian target of rapamycin
NGS: Next generation sequencing
PEComas: Perivascular epithelioid cell tumor
TSC: Tuberous sclerosis complex syndrome

## References

[1] Fujii Y, Ajima J, Oka K, Tosaka A, Takehara Y (1995). Benign renal tumors detected among healthy adults by abdominal ultrasonography. Eur Urol.

[2] Fittschen A (2014). Prevalence of sporadic renal angiomyolipoma: a retrospective analysis of 61,389 in- and out-patients. Abdom Imaging.

[3] Lienert AR, Nicol D (2012). Renal angiomyolipoma. BJU Int.

[4] He W (2013). Epithelioid angiomyolipoma of the kidney: pathological features and clinical outcome in a series of consecutively resected tumors. Mod Pathol.

[5] Faraji H, Nguyen BN, Mai KT (2009). Renal epithelioid angiomyolipoma: a study of six cases and a meta-analytic study. Development of criteria for screening the entity with prognostic significance. Histopathology.

[6] Brimo F (2010). Renal epithelioid angiomyolipoma with atypia: a series of 40 cases with emphasis on clinicopathologic prognostic indicators of malignancy. Am J Surg Pathol.

[7] Nese N (2011). Pure epithelioid PEComas (so-called epithelioid angiomyolipoma) of the kidney: a clinicopathologic study of 41 cases: detailed assessment of morphology and risk stratification. Am J Surg Pathol.

[8] Kwiatkowski DJ (2003). Tuberous sclerosis: from tubers to mTOR. Ann Hum Genet.

[9] Saxton RA, Sabatini DM (2017). mTOR signaling in growth, metabolism, and disease. Cell.

[10] Franz DN (2016). Long-term use of Everolimus in patients with tuberous sclerosis complex: final results from the EXIST-1 study. PLoS One.

[11] Wagner AJ (2010). Clinical activity of mTOR inhibition with sirolimus in malignant perivascular epithelioid cell tumors: targeting the pathogenic activation of mTORC1 in tumors. J Clin Oncol.

[12] Dickson MA, Schwartz GK, Antonescu CR, Kwiatkowski DJ, Malinowska IA (2013). Extrarenal perivascular epithelioid cell tumors (PEComas) respond to mTOR inhibition: clinical and molecular correlates. Int J Cancer.

[13] Wolff N (2010). Sirolimus and temsirolimus for epithelioid angiomyolipoma. J Clin Oncol.

[14] Kohno J (2013). Role of mammalian target of rapamycin inhibitor in the treatment of metastatic epithelioid angiomyolipoma: a case report. Int J Urol.

[15] Wyluda E, Baquero G, Lamparella N, Abendroth C, Drabick J (2013). Fatal malignant metastastic epithelioid angiomyolipoma presenting in a young woman: case report and review of the literature. Rare Tumors.

[16] Higa F (2009). Malignant epithelioid angiomyolipoma in the kidney and liver of a patient with pulmonary lymphangioleiomyomatosis: lack of response to sirolimus. Intern Med.

[17] Iyer G (2012). Genome sequencing identifies a basis for everolimus sensitivity. Science.

[18] Wagle N (2014). Activating mTOR mutations in a patient with an extraordinary response on a phase I trial of everolimus and pazopanib. Cancer Discov.

[19] Wagle N (2014). Response and acquired resistance to everolimus in anaplastic thyroid cancer. N Engl J Med.

[20] Kwiatkowski DJ, et al. Mutations in TSC1, TSC2, and MTOR are associated with response to rapalogs in patients with metastatic renal cell carcinoma. Clin Cancer Res. 2016; 10.1158/1078-0432.CCR-15-2631 PMC4976069 26831717

[21] Henske EP (1995). Loss of heterozygosity in the tuberous sclerosis (TSC2) region of chromosome band 16p13 occurs in sporadic as well as TSC-associated renal angiomyolipomas. Genes Chromosomes Cancer.

[22] Qin W (2011). Angiomyolipoma have common mutations in TSC2 but no other common genetic events. PLoS One.

[23] Giannikou K (2016). Whole exome sequencing identifies TSC1/TSC2 Biallelic loss as the primary and sufficient driver event for renal Angiomyolipoma development. PLoS Genet.

[24] Pan CC (2008). Constant allelic alteration on chromosome 16p (TSC2 gene) in perivascular epithelioid cell tumour (PEComa): genetic evidence for the relationship of PEComa with angiomyolipoma. J Pathol.

[25] Guo B, Song H, Yue J, Li G (2016). Malignant renal epithelioid angiomyolipoma: a case report and review of the literature. Oncol Lett.

[26] Citak EC (2015). Malignant epitheloid angiomyolipoma of the kidney in a child treated with sunitinib, everolimus and axitinib. Can Urol Assoc J.

[27] Kenerson H, Folpe AL, Takayama TK, Yeung RS (2007). Activation of the mTOR pathway in sporadic angiomyolipomas and other perivascular epithelioid cell neoplasms. Hum Pathol.

[28] Bissler JJ (2013). Everolimus for angiomyolipoma associated with tuberous sclerosis complex or sporadic lymphangioleiomyomatosis (EXIST-2): a multicentre, randomised, double-blind, placebo-controlled trial. Lancet.

[29] Nagashima Y (2013). Editorial comment to role of mammalian target of rapamycin inhibitor in the treatment of metastatic epithelioid angiomyolipoma: a case report. Int J Urol.

[30] Gennatas C, Michalaki V, Kairi PV, Kondi-Paphiti A, Voros D (2012). Successful treatment with the mTOR inhibitor everolimus in a patient with perivascular epithelioid cell tumor. World J Surg Oncol.

[31] Shitara K (2011). Dramatic tumor response to everolimus for malignant epithelioid angiomyolipoma. Jpn J Clin Oncol.

